# Spontaneous Subarachnoid Hemorrhage in a Patient with a Co-Existent Posterior Communicating Artery Aneurysm and Cervical Spine Aneurysm Associated with Ventral Arterio-Venous Fistula

**DOI:** 10.3390/brainsci10020070

**Published:** 2020-01-28

**Authors:** Aleš Hejčl, Jan Lodin, Filip Cihlář, Martin Sameš

**Affiliations:** 1 Department of Neurosurgery, J.E. Purkinje University, Masaryk Hospital, Sociální péče 12A, 40113 Ústí nad Labem, Czech Republicmartin.sames@kzcr.eu (M.S.); 2 International Clinical Research Center, St. Anne’s University Hospital, 38975 Brno, Czech Republic; 3 Institute of Experimental Medicine, Academy of Sciences of the Czech Republic, Vídeňská 1083, 14220 Prague, Czech Republic; 4 Department of Radiology, J. E. Purkinje University, Masaryk Hospital, 40113 Ústí nad Labem, Czech Republic; filip.cihlar@kzcr.eu

**Keywords:** subarachnoid hemorrhage, spinal AV fistula, aneurysm, hydrocephalus

## Abstract

Severe spontaneous subarachnoid hemorrhage (SAH) is predominantly caused by aneurysm rupture, with non-aneurysmal vascular lesions representing only a minority of possible causes. We present the case of a 58-year old lady with a coincidental posterior communicating artery (PCom) aneurysm and a high cervical spine arterio-venous fistula associated with a small ruptured aneurysm. After the emergency clipping of the PCom aneurysm, additional diagnostic procedures—repeated digital subtraction angiography and spinal magnetic resonance imaging, revealed the actual cause of the SAH, a type-A ventral intradural fistula at cervical level C2/3. The fistula was treated micro surgically via a ventral approach using C3 somatectomy and C2-4 stabilization after the initial failure of endovascular therapy. Furthermore, the patient was treated for complications associated with severe SAH, including acute hydrocephalus and meningitis. In cases where the SAH pattern and perioperative findings do not suggest an intracranial aneurysm as the source of SAH, further diagnostic investigation is warranted to discover the real cause. Patients with severe non-aneurysmal SAH require a similar algorithm in diagnosing the cause of the hemorrhage as well as complex conditions such as ruptured aneurysms.

## 1. Introduction

Spontaneous subarachnoid hemorrhage (SAH) is caused by an intracranial aneurysm rupture in 85% of cases [1]. Concurrently, there has been an increasing incidence of patients with non-aneurysmal SAH [2]. Non-aneurysmal SAH can be divided into perimesencephalic SAH (PMSAH) and non-perimesencephalic SAH (non-PMSAH). While patients with PMSAH, in general, seem to have good outcomes with a minimal chance of an undisclosed lesion diagnosis on initial digital subtraction angiography (DSA), about 12% of patients with non-PMSAH have a cranial or spinal lesion responsible for the SAH [3]. Spinal arterio-venous fistulas (AVFs) are rare lesions, which usually present with pain, paralysis, or paresthesias, however, about 9% may also present with SAH [4]. In this study, we present the case of a patient who suffered severe SAH and harbored a ruptured ventral intradural cervical AVF with an associated aneurysm and a coincidental, right posterior communicating artery (PCom) aneurysm. We demonstrate our diagnostic approach and treatment of the lesion itself as well as the complications associated with SAH in such a rare case.

## 2. Case Report

**Figure 1:**
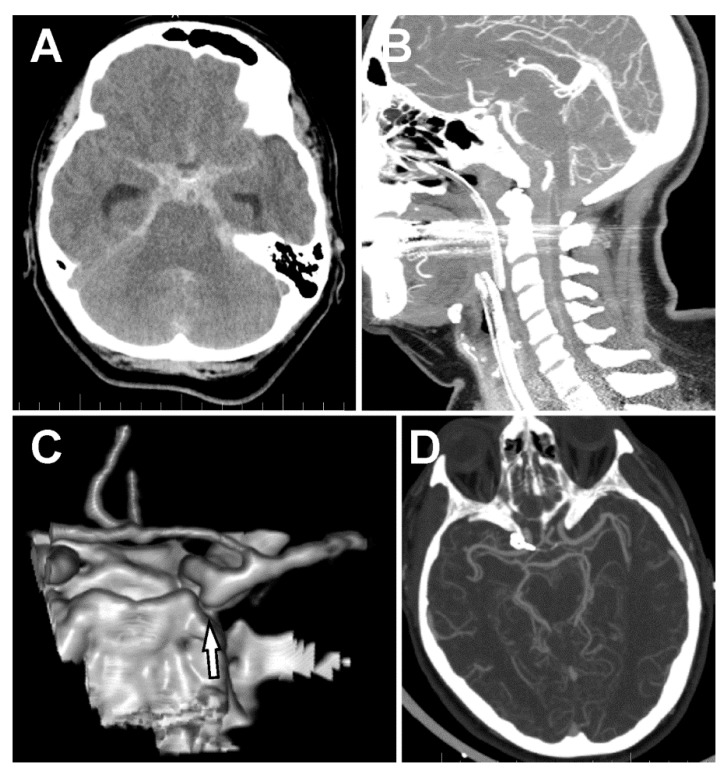
Subarachnoid hemorrhage and the posterior communicating artery (PCom) aneurysm. (**A**) Massive spontaneous subarachnoid hemorrhage (SAH) in the basal cisterns and both Sylvian cisterns. (**B**) SAH in front of the brain stem and the ventral subarachnoid space all the way down to C6. (**C**) 3D-CT angiography (CTA) showing an aneurysm of the right PCom artery directing downward to the posterior fossa. (**D**) CTA after clipping of the PCom artery.

A 58-year old nurse experienced a sudden severe nuchal headache at home. After an ambulance arrived, she lost consciousness (Glasgow Coma Scale, GCS 7). She was intubated, sedated, and transferred to the local hospital. The emergency computed tomography (CT) scan showed a massive subarachnoid hemorrhage predominantly located in the basal cisterns, posterior fossa (pretruncal), and in the ventral spinal canal down to level C5 (Figure 1A,B). CT angiography (CTA) showed an aneurysm located on the PCom with an inferiorly directed sac and no other source of hemorrhage (Figure 1C). The patient was transferred to our hospital, after necessary preparation at the Emergency Department she was taken acutely into the operating room, where extra ventricular drainage (EVD) was implanted, and the aneurysm was secured with two stacked, slightly curved 5 mm clips from a right lateral supraorbital craniotomy [5,6]. However, further inspection of the aneurysm under the operating microscope showed no signs of its rupture. The patient was extubated the following day, and a postoperative CT showed no signs of complications (hemorrhage, ischemia) as well as confirming complete exclusion of the PCom aneurysm (Figure 1D). Due to the massive subarachnoid hemorrhage in front of the brain stem, with significant extension in the spinal canal and no clear sign of aneurysm rupture during surgery, we performed cerebral DSA on the third day after clipping. Angiography confirmed the complete exclusion of the aneurysm with no other source of hemorrhage. Still, we were not satisfied with the diagnostic result and therefore, planned a check-up angiography three weeks after the initial SAH. During the initial 14 days following hemorrhage, the patient was monitored at the Intensive Care Unit. The patient was extubated on the fourth day after surgery. As the patient was dependent on the EVD, we exchanged the EVD on Day 9 for a lumbar drain. As the patient repeatedly proved to be drain-dependent, we were forced to implant a ventriculo-peritoneal (VP) shunt. Unfortunately, two revisions of the shunt were necessary due to its malposition the following day. We removed the VP shunt 4 days later because the patient contracted meningitis despite antibiotic therapy. The patient was monitored for cerebral vasospasms with repeated transcranial dopplerometry (TCD). No vasospasms were detected.

**Figure 2:**
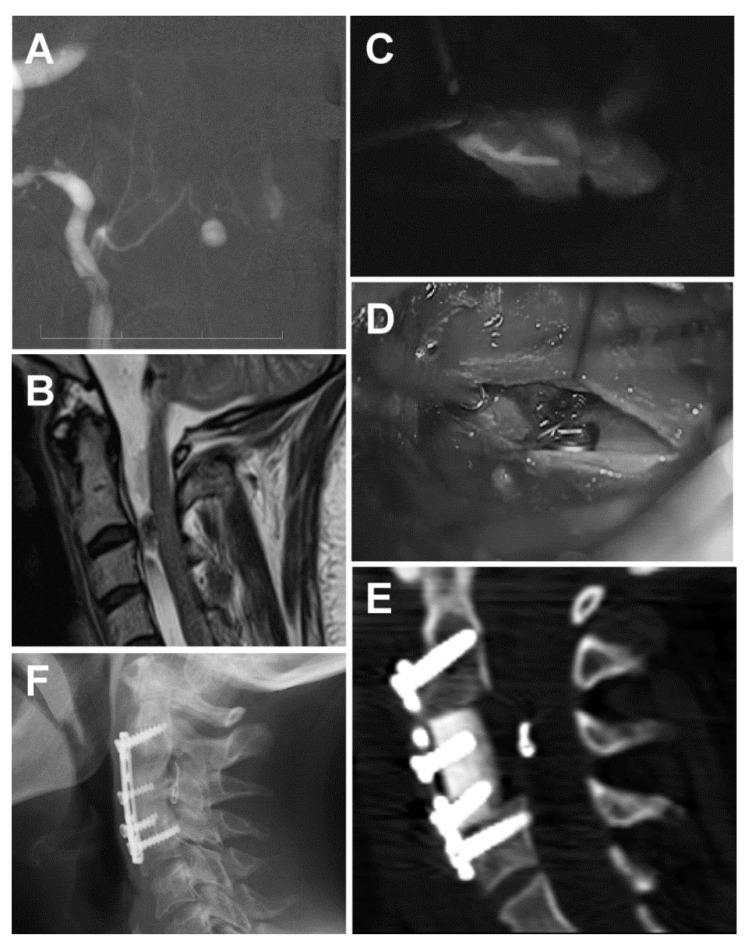
Spinal aneurysm associated with a ventral cervical arterio-venous (AV) fistula (**A**) AV fistula associated with an aneurysm displayed with a super-selective digital subtraction angiography (DSA). (**B**) Sagittal T2W MRI showing a lesion ventral to the spinal cord at the C2/3 level. (**C**) Perioperative indocyanine green (ICG) showing the pedicle feeding a type IVA ventral AV fistula from the anterior spinal artery. (**D**) Perioperative microscopic image after clipping the AV fistula. (**E**) Tibial bone allograft with a titanium plate and bicortical screws after C3 somatectomy. The clip is visible in front of the spinal cord, just in the middle of the approach. (**F**) The X-ray image shows the complete fusion of the allograft with the cervical bodies.

The follow-up angiography revealed a small ventral arterio-venous fistula associated with a small aneurysm at cervical level C2/3. It was a type III AV fistula, according to Kim and Spetzler [7], and a type IVa based on the classification of Heros [8] (Figure 2A). It was fed by a sulcal artery from the anterior spinal artery (ASA). A subsequent cervical spine MRI confirmed a left paramedian lesion ventral to the spinal cord at the level of the C2/3 intervertebral disc (Figure 2B). The patient was prepared for an endovascular procedure. The right vertebral artery was catheterized up to the radicular branch. Further selective catheterization was performed up to the midline. However, further super-selective catheterization did not allow passage through the sulcal artery. Therefore, the endovascular procedure was terminated, and a surgical procedure was planned and performed 6 days later.

The lesion was approached via a C3 somatectomy. Surgery was performed with the aid of electrophysiological monitoring (motor evoked potentials, MEPs). After a midline durotomy in the vertical direction, we identified the anterior spinal artery and the right radicular artery of C2 with the aneurysm just left of the midline. Dissection of the arteries was very difficult due to the scarring of the arachnoid, which was yellowish as a result of the SAH. Using indocyanine green (ICG), we identified the pedicle and applied a clip (Figure 2C,D). Using a micro-Doppler and ICG, we confirmed the complete exclusion of the fistula. MEPs were stable throughout the procedure. We used an allograft with a titanium plate and bicortical screws to stabilize the cervical spine (Figure 2E). The postoperative course was uneventful. A follow-up lumbar puncture showed normal pressure, and the patient was improving; therefore, there was no need for further shunt surgery. The patient was transferred to a physical therapy department. Nine months after the SAH, the patient complained only of slight vertigo and right side hypacusis. An X-ray 20 months after the surgery showed complete C3–C5 fusion (Figure 2F). The patient showed no further symptoms of hydrocephalus, and her ventricles remained narrow on follow-up CT scans.

## 3. Discussion

Aneurysmal SAH is associated with high mortality and morbidity [1,9]. However, in about 15% of cases, no vascular intracranial lesion is discovered on initial DSA. Non-aneurysmal non-PMSAH tend to have a similar clinical course to an aneurysmal SAH and have a significant risk of an underlying cause of hemorrhage [10]. Up to 2004, there have been 17 cases of spinal AVFs associated with SAH.

In our case, the patient presented with massive posterior fossa SAH and hemocephalus due to a ruptured aneurysm associated with a ventral cervical spine fistula. However, only a PCom aneurysm was diagnosed on the initial emergency CT angiography and was mistakenly considered to be the source of bleeding. A similar scenario was described by Vates et al. [11]. However, perioperative findings suggested that the aneurysm was unruptured (no thrombus or brain adherence to the sac, irregularities of the sac, or translucent aneurysm wall) blood from the SAH extended far down the spinal canal. This led to further diagnostic procedures, including repeated DSAs. Akcakaya et al. found that there is approximately a 5% chance of diagnosing an underlying cause (aneurysm) of SAH on subsequent DSA. The same authors used spinal MRIs in patients with non-aSAH after the second DSA was negative. In such cases, they present positive findings on spinal MRIs in 3.7% of cases between 2 weeks to 12 months after the SAH [3]. Germans et al. do not recommend routine radiological investigation of the spinal axis of patients with non-aneurysmal SAH [12]. However, in our case, retrospectively, the distribution of SAH was indicative of a spinal lesion (Figure 1B), and the MRI could have perhaps been performed earlier, even after the first DSA.

The cause of SAH was an aneurysm associated with an AV fistula at the cervical C23 level. Several case reports and miniseries describe patients with ruptured AV fistulas who presented with SAH. They are summarized in Table 1. Our case was a ventral intradural AV fistula associated with an aneurysm, type IV, based on the classical Heros classification or type III fistula, according to the modified classification of Kim and Spetzler [7,8]. Ventral AV fistulas are very rare, accounting for only 6% of all AVFs. They predominantly occur in younger patients and are most often found at the conus medullaris and cauda equina. Our patient was 58 years old at the time of presentation, and the lesion was located in the cervical level. Koch et al. presented a very similar case, in which the SAH was caused by an AV fistula at the L4 level [13]. Hemorrhage is more common (20–40%) in these AVFs due to a higher flow rate. In rare cases, the aneurysm may even be intramedullary, and, in case of rupture, may cause an intramedullary hematoma [14]. Ventral AVFs can be divided into three subtypes: A, B, and C. Our patient harbored an A type of fistula, which is characterized by a single low flow shunt with characteristics similar to dorsal intradural AVFs. In our case, the AVF was associated with an aneurysm, the cause of the SAH. Both surgical and endovascular treatments have been used for the treatment of these fistulas, as shown in Table 1. The type of treatment has usually been made after a detailed analysis of the angioarchitecture of the vascular lesion. Microsurgery is the treatment of choice in the subtypes A and B because EVT is often limited by ASA involvement, which is difficult to catheterize and navigate [15,16]. This is exactly what we experienced in our case during the EVT procedure. Concerning the surgical approach, the location of the aneurysm and the fistula required a C3 somatectomy to gain the space required for proper dissection of the lesion. This approach for a ventral AVF was probably first described by Hida et al. [16] DSA, together with MRI, were very helpful for planning the approach. The dural opening was in the midline above the anterior spinal artery. Perioperative ICG proved to be a useful tool for demarcation and identification of the fistula and aneurysm as the post-hemorrhage scarring and coloring around the lesion made dissection difficult. ICG was later also used to confirm the patency of the ASA after clipping the feeder. ICG with different flow mapping may be even more essential for complex spinal AVMs [4]. Another alternative may be perioperative DSA, as advocated by Ogawa et al. [17]. During surgery, we made sure that the ASA was preserved [7]. The procedure was performed under MEPs monitoring, which we consider a necessity for performing safe surgery of spinal AVMs.

The main disadvantage of the ventral approach is the necessity of resecting one or more vertebral bodies. In our case, only a one-level somatectomy was required to obtain an adequate approach, due to small fistula size. Markert et al. propose an alternative, an extreme lateral approach to reach ventral AV lesions without the need for resecting vertebral bodies [18]. However, they described this approach for a fistula at the C1/2 level, which would require a transoral route for the ventral approach. This would be associated with an even higher risk of cerebrospinal fluid (CSF) leak and infection. The ventral approach, in our case, was elegant, and the allograft fused very well without any functional deficit for the patient. For larger lesions requiring multiple somatectomies or lesions located at the levels with a more complicated ventral approach (upper thoracic segments), the extreme lateral approach is a reasonable alternative. Further technological developments, such as in neuroendoscopy, may provide additional tools for obtaining an excellent view as well as adequate space via a minimally invasive approach [17].

Non-aneurysmal non-PMSAH present with similar complications as aneurysmal SAH. In our case, the patient suffered from early hydrocephalus. In patients with non-aneurysmal SAH, the risk of early hydrocephalus is around 30%, with 10% eventually requiring shunt placement [19]. According to these authors, early hydrocephalus was also associated with an unfavorable outcome. In our case, the patient suffered from acute hydrocephalus and required repeated CSF shunting. Throughout the treatment, she experienced some typical complications associated with CSF drainage, such as malposition with revisions, eventually leading to meningitis and shunt removal. Fortunately, the hydrocephalus resolved spontaneously without causing any additional morbidity.

## 4. Conclusions

Spinal AV fistulas are rare causes of SAH. Accurate diagnosis and microsurgery are the keys to successful treatment of this type of a ventral AV fistula, while risk factors associated with this severe non-aneurysmal SAH should be anticipated and treated to improve the probability of a good outcome in these patients.

## Figures and Tables

**Table 1:** Literature summary of spinal arterio-venous (AV) fistulas which presented with spontaneous subarachnoid hemorrhage (SAH).

Author	Year	Patient Age	Spine Level	Outcome	Treatment
Inoue et al. [20]	2019	59	High Cervical	mRS5	refused treatment
Liu et al. [21]	2008	26	High Cervical	mRS0	surgical resection
Akter et al. [22]	2011	68	High Cervical	N/A	surgical resection
Akter et al.	2011	53	High Cervical	N/A	surgical resection
Akter et al.	2011	56	Cervical	N/A	surgical resection
Akter et al.	2011	60	Thoracic	N/A	surgical resection
Hayashi et al. [23]	2004	67	Cervical	mRS0	embolization
Kai et al. [24]	2005	54	High Cervical	mRS1	surgical resection
Kai et al.	2005	56	High Cervical	mRS1	surgical resection
Fernandéz et al. [25]	2008	5	High Cervical	mRS1	surgical resection
Poisson et al. [26]	2008	8 months	Low thoracic	mRS3	embolization
Lv et al. [27]	2012	17	High Cervical	mRS2	embolization
Ohmori et al. [28]	2005	42	Low thoracic	mRS0	surgical resection
Bagherpour et al. [29]	2016	14	Conus medullaris	mRS0	surgical resection
Ohba et al. [30]	2011	55	High Cervical	mRS1	surgical resection
Vates et al. [11]	2001	65	Conus medullaris	mRS4	surgical resection
Kominami et al. [31]	1996	12	High Cervical	N/A	embolization
Alshekhlee et al. [32]	2011	57	High Cervical	mRS4	embolization
Hida et al. [16]	2002	58	High Cervical	N/A	surgical resection
Hida et al.	2002	59	High Cervical	N/A	surgical resection
Hida et al.	2002	62	High Cervical	N/A	surgical resection
Hida et al.	2002	62	Low Cervical	N/A	surgical resection
Hida et al.	2002	34	Low Cervical	N/A	combination
Wakai et al. [33]	1992	8	Low Cervical	mRS1	surgical resection
